# Guided Imagery And Progressive Muscle Relaxation as a Cluster of Symptoms Management Intervention in Patients Receiving Chemotherapy: A Randomized Control Trial

**DOI:** 10.1371/journal.pone.0156911

**Published:** 2016-06-24

**Authors:** Andreas Charalambous, Margarita Giannakopoulou, Evaggelos Bozas, Yiola Marcou, Petros Kitsios, Lefkios Paikousis

**Affiliations:** 1 Cyprus University of Technology, Limassol, Cyprus; 2 University of Turku, Turku, Finland; 3 University of Athens, Athens, Greece; 4 Bank of Cyprus Oncology Centre, Nicosia, Cyprus; 5 Improvast Analytical Services Company, Nicosia, Cyprus; ACTREC (Advanced Centre for Treatment, Research and Education in Cancer) / Tata Memorial Centre, INDIA

## Abstract

**Objective:**

Patients receiving chemotherapy often experience many different symptoms that can be difficult to alleviate and ultimately negatively influence their quality of life. Such symptoms include pain, fatigue, nausea, vomiting and retching, anxiety and depression. There is a gap in the relevant literature on the effectiveness of cognitive-behavioural and relaxation techniques in symptom clusters. The study reflects this gap in the literature and aimed to test the effectiveness of Guided Imagery (GI) and Progressive Muscle Relaxation (PMR) on a cluster of symptoms experienced by patients undergoing chemotherapy.

**Methods:**

This was a randomized control trial with 208 patients equally assigned either in the intervention or the control group. Measurements in both groups were collected at baseline and at completion of intervention (4 weeks). Patients were assessed for pain, fatigue, nausea, vomiting and retching, anxiety and depression. The overall management of the cluster was also assessed based on the patients’ self-reported health related quality of life-HRQoL. Chi-square tests (X^2^), independent T-tests and Linear Mixed Models were calculated.

**Results:**

Patients in the intervention group experienced lower levels of *Fatigue* (p<0.0.0225), and *Pain* (p = 0.0003) compared to those in the control group and experienced better *HRQoL* (p<0.0001) [PRE-POST: Intervention: *Pain* 4.2(2.5) - 2.5(1.6), *Fatigue* 27.6(4.1) - 19.3(4.1), *HRQoL* 54.9(22.7) - 64.5(23), Control: *Pain* 3.5(1.7) - 4.8(1.5), *Fatigue* 28.7(4.1) - 32.5(3.8), *HRQoL* 51.9(22.3)– 41.2(24.1)]. Nausea, vomiting and retching occurred significantly less often in the intervention group [pre-post: 25.4(5.9)– 20.6(5.6) compared to the control group (17.8(6.5)– 22.7(5.3) (F = 58.50 p<0.0001). More patients in the control group (pre:n = 33-post:n = 47) were found to be moderately depressed compared to those in the intervention group (pre:n = 35-post:n = 15) (X^2^ = 5.93; p = 0.02).

**Conclusion:**

This study provided evidence that the combination of GI and PMR can be effective in the management of a cluster of symptoms in cancer patients receiving chemotherapy. These techniques can complement existing management measures to achieve a comprehensive management of this symptom cluster and increase patients HRQoL.

**Trial Registration:**

ClinicalTrials.gov NCT01275872

## Background

The relevant literature reveals that the mainstream of research on symptoms has been focused either on a single symptom, such as pain or fatigue, or on their associated symptoms, such as depression or anxiety [1]. The experience of concurrent symptoms (cluster) in cancer patient groups receiving chemotherapy and/or radiotherapy is not an uncommon finding in the literature [2–4]. A symptom cluster is a condition where two or more symptoms that are related to each other occur simultaneously. Symptom clustering is a method for grouping together multiple symptoms which occur with disease and its treatment [5]. Although the preceding studies have contributed to the better understanding of some symptoms, they do not offer much information in the cases where the patient experiences a cluster of symptoms and healthcare professionals are called upon to deal with the complexity caused by this situation in clinical practice.

Much has been written about symptom clusters [6], but very little in the comprehensive management of these on a clinical level care [7]. Breast and prostate cancer are among the most common, and patients with these malignancies often experience various symptom clusters before [7], during or/and after treatment [5]. The significant improvement in the survival rates recorded in these groups, means that more people live longer and have to deal with the symptom cluster more frequently and for longer periods of time. Furthermore, the introduction of new therapies was accompanied by the emergence of new, persistent and debilating symptom clusters [5,7].

This paper will focus in the reporting of research data in relation to the effective management of symptom clusters through the implementation of Complementary and Alternative methods (CAM) including cognitive-behavioral interventions in patients with breast cancer and prostate cancer. Preceding studies and systematic reviews of cognitive-behavioral strategies including guided imagery and relaxation have revealed their positive effect in reducing cancer pain, nausea and vomiting. However, these studies primarily tested the effectiveness of these strategies solely on one symptom [8]. Therefore, their effect (if any) on the symptom clusters remains unknown and unexplored in the relevant literature.

The overall aim of this study was to explore whether the combination of Guided Imagery (GI) and Progressive Muscle Relaxation (PMR) can be effective on a cluster of symptoms experienced by patients diagnosed with breast or prostate cancer undergoing chemotherapy.

The study hypothesises that the patients in the intervention group will experience lower levels of fatigue, pain, nausea, vomiting and retching, anxiety, depression and thus higher levels of Health Related Quality of Life (HRQoL) compared to those in the control group.

## Methods

### Ethical considerations

This study was in compliance with the Declaration of Helsinki and the protocol was approved by the Cyprus National Bioethics Committee (I.D. CNBC/EP/2010/06) on 22^nd^ June 2010. All participants signed an informed consent prior to their participation in the study.

### Design

This was a randomised control parallel design trial with two groups. The study was fully funded by the Cyprus University of Technology (INT-480619-2010). The funder did not have influence over the trial design or the reporting data. The clinicaltrials.gov identifier is NCT01275872. The study was registered in January 2011, although it officially started in 2010. Issues with releasing the funds and unanticipated difficulties in patient recruitment delayed the registration. The study was registered within 21 days from the recruitment of the first participant. The authors confirm that all ongoing and related trials for this intervention are registered.

#### Randomisation and masking

Patients who consented to take part in the study were randomised in a 1:1 ratio to either the intervention or the control group using a computer-based minimisation algorithm stratifying for cancer type and the cancer centres. This was done by an independent third party external to the study. Due to practicality reasons the participants were not masked to the allocated intervention consisting of GI and PMR. Patients’ carers, the members of the research team and the outcome assessors were blinded by the assigning of unique identification numbers from the external party.

### Settings

Patients were assessed based on the study’s inclusion and exclusion criteria by their oncologists and referred to the study. Referrals took place at the out-patient clinics of the three participating cancer care centres. Patients’ intervention and assessments were undertaken at their home based on their preferences.

### Sample size

Type I error, power, assumptions on response rate and standard deviation, and expected treatment effect were taken into consideration in calculating sample size. The type I error and power were set at conventional levels (5% for two-sided type I error and 80% for power). Assumptions were based on preceding studies’ available data and published results. Based on these, slightly more than 25% was added in the final calculated sample to ensure that the size was large enough to detect any effect after possible low response rates, drop-outs, refusals and losses in follow-ups. We aimed in detecting a meaningful clinical effect i.e. changes of >10 points on the GQOL/QOL scale of the QLQ-C30 are considered meaningful in longitudinal studies [9]. Using G-power software for independent samples t-test for detecting a 10 mean difference with an SD = 25 for each group, we established a minimum of 100 sample size for each group. These parameters yield a small to medium effect size (d = 0.40) with an actual power of 0.803. The above configuration of the sample size, also satisfies power analysis of a paired t-test with the same effect size (d = 0.40) and a mean change of 10 points in the QLQ questionnaires. Therefore, the overall sample size needed to be set at 200 and this was increased to 256 as to accommodate all the assumptions discussed above. The final sample size differs slightly (8 more patients included) compared to the one reported in the study’s protocol.

### Participants

**Fig 1:**
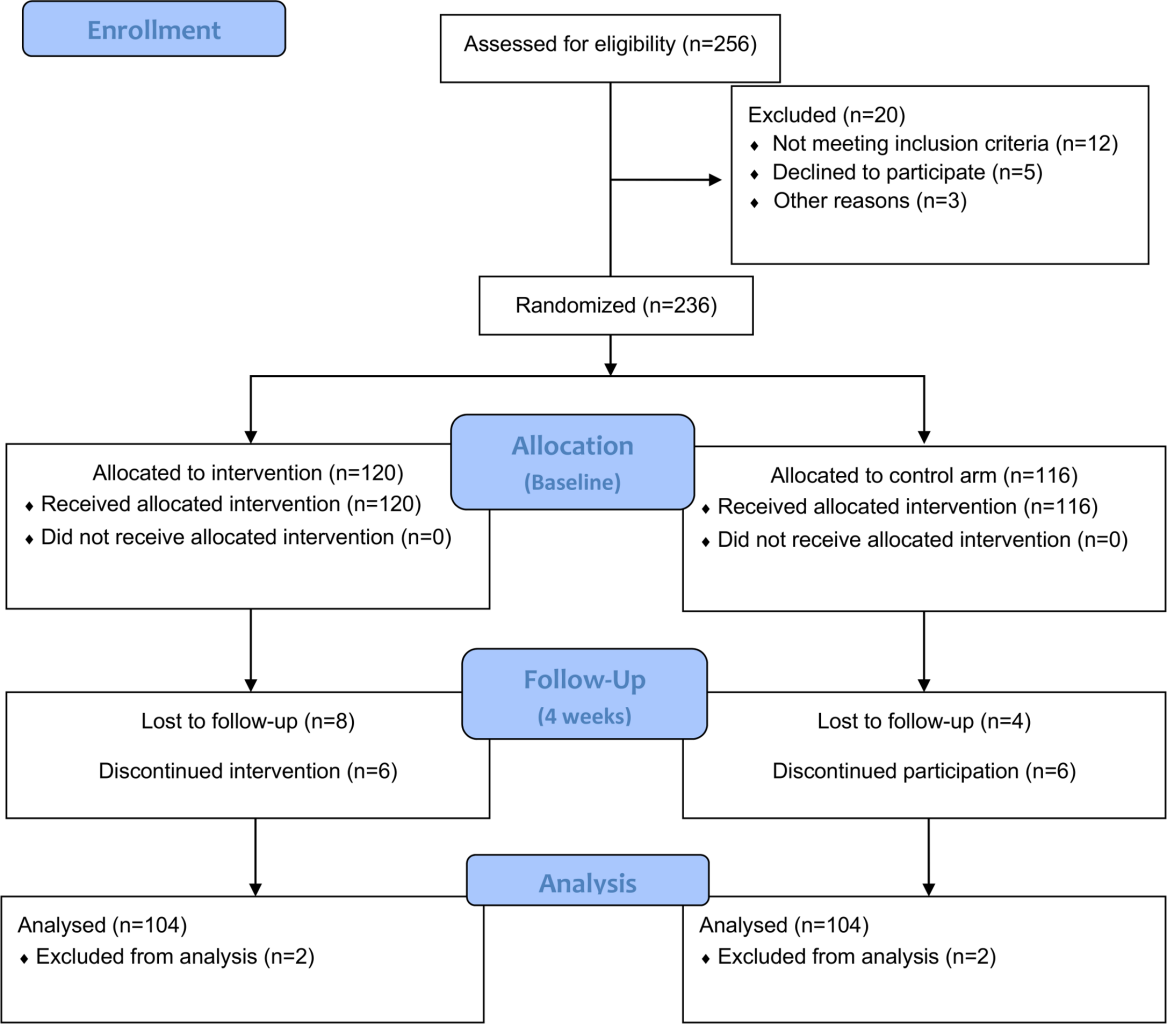
CONSORT Flow Diagram.

Patients were assessed for their eligibility on the following criteria: (a) clinical diagnosis of breast (clinical stage T3N1M0) or prostate cancer (clinical stage T3a, Gleason score ≥ 8), (b) receiving chemotherapy, (c) experience of fatigue, pain, nausea and vomiting, anxiety, depression (d) able to follow instructions, (e) good cognitive ability, and (f) willingness to participate. Patients with visual and/or hearing impairment and/or cognitive impairment, xerostomia and/or oral mucositis (as nausea and vomiting exacerbating factors) were excluded from the study. In the period between January 2011 and December 2012, 256 patients in total were assessed for eligibility and data from 208 patients were completed and included in the analysis (Fig 1).

### Intervention and procedures

The initial study protocol entailed that the patients will only experience 3 supervised sessions of GI and PMR. However, the research team decided that in order to increase the effectiveness of the intervention one more supervised session should be added in addition to the unsupervised ones. Therefore the patients experienced 4 weekly supervised and daily unsupervised sessions of GI and PMR based on a script that was fortified with auditory, tactile and olfactory images and accompanied by soft music camouflaged with alpha waves pulses [10, 11]. The intervention included a 2-minute breathing exercise, followed by a 10-minute progressive muscle relaxation exercise and a 15-minute pleasant guided imagery session. The control group only received the usual (standardised) treatment as this is described in international guidelines for each of the reported symptoms [i.e for anxiety and depression weekly meetings with centre’s psychologist, for nausea, vomiting and retching patients were treated on the basis of the MASCC/ESMO Antiemetic Guideline, management of cancer pain was based on the ESMO Clinical Practice Guidelines and fatigue was managed on the basis of the NCCN Practice Guidelines for Cancer-Related Fatigue (excluding any Complementary and Alternative Therapies)].

#### Guided imagery

Guided imagery is the use of mental visualization (mental images) to improve mood and physical well-being. A mental image can be defined as “a thought with sensory qualities.” It is something we mentally see, hear, taste, smell, touch, or feel [12]. The term guided imagery refers to a wide variety of techniques; however, for the purpose of this study the researchers have employed simple visualization and direct suggestion using imagery. The connection between the mind and physical health has been well documented and extensively studied in previous studies [12]. Positive mental imagery has been used extensively in cancer care and has been found effective in various situations such as to promote relaxation and reduce stress [13], improve mood [11], alleviate pain [14–16], boost the immune system [17], and minimise nausea and vomiting [18].

#### Progressive Muscle Relaxation

Progressive Muscle Relaxation is a nursing intervention from the Nursing Interventions Classification (NIC) defined as facilitating the tensing and releasing of successive muscle groups while attending to the resulting differences in sensation [19]. Through this repetitive practice the patient is able to quickly learn how to recognize and distinguish the associated feelings of a tensed muscle and a completely relaxed muscle. Possessing this simple knowledge, one can then induce physical muscular relaxation at the first signs of tension accompanied by anxiety. The accomplishment of physical relaxation promotes mental calmness in a parallel manner. Progressive muscle relaxation has also been used in cancer care and has been found effective among other in pain [20], fatigue [21], nausea and vomiting [22] and anxiety [23].

### Assessments

Several assessments were performed as part of the study that reflected the patients’ experienced symptoms. These included pain, cancer related fatigue, anxiety, depression, nausea, vomiting and retching. Patients’ Health Related Quality of Life (HRQoL) was assessed as an indicator of better symptom cluster management and as a result of the tested intervention. Patients in both groups were assessed on the symptoms above at baseline (prior the intervention) and at the end of the intervention (at 4 weeks).

#### Pain

The level of pain was assessed with the use of a numeric pain scale. The scale asked the patients to rate their pain level on a 10-point numeric scale where 0 denoted the absence of pain and 10 denoted the worst experienced level of pain.

#### Fatigue

The Cancer Fatigue Scale (CFS) measures total fatigue score ranging from 0 (lowest level) to 60 (highest level). The CFS is a three-dimensional inventory of 15 items which was originally developed in Japan [24]. The consisting 3 subscales assess patients’ responses on physical, affective, and cognitive aspects of their daily living. Each item is rated on a scale of 1 (= not at all) to 5 (= very much) and patients are asked to circle the number that describes their current state. The possible scores range from 0 to 28 for the physical, 0 to 16 for the affective, and 0 to 16 for the cognitive subscale.

#### Nausea, vomiting and retching

The Revised Rhodes index of nausea, vomiting and retching (INVR) is an instrument consisting of eight 5-point self-reported items designed to assess subjective and objective factors of nausea, vomiting and retching in various situations [25, 26]. Several preceding studies demonstrated the validity and reliability of the INVR for cancer patients [27, 28]. Subscale scores can be used to calculate nausea, vomiting and retching experience, occurrence and distress separately as well as scores for total experience, occurrence and distress. Scores for individual items can range from 0 to 4 with higher scores indicating more nausea, vomiting or retching.

#### Zung self-rating anxiety scale SAS

The degree of anxiety was measured by the Zung self-rating anxiety scale. The SAS is a self-reported questionnaire with 20 items rated on a four-point scale in relation to whether the person has experienced each specific symptom ‘‘none or a little of the time” (rating 1), ‘‘some of the time” (2), ‘‘a good part of the time” (3), or ‘‘most or all of the time” (4). The total SAS score may vary from 20 (no anxiety at all) to 80 (severe anxiety). A high score indicated a high degree of anxiety. Zung set a cutoff point at a raw score of 36, in which he described participants as having anxiety that ‘was clinically significant” (see page 18 in [29], although this does not equate to an Anxiety Disorder according to DSM criteria.

#### Beck Depression Inventory-II

The BDI-II is a self-reported inventory that assesses the presence and severity of depressive symptoms experienced by the patients. The BDI-II includes 21 items, with each item consisting of four statements reflecting varying degrees of symptom severity. Respondents were instructed to circle the number (ranging from 0 to 3) that corresponds with the statement that best applies to them. A rating of 0 indicates the absence of a symptom, while a rating of 3 is indicative of a severe symptom. Ratings from the 21 items are summed to calculate a total score, which can range from 0 to 63. According to Beck et al. [30], scores of 0–13 are suggestive of minimal depression, scores of 14–19 are indicative of mild depression, scores of 20–28 are indicative of moderate depression, and scores of 29 or greater are suggestive of severe depression.

#### Health related Quality of Life

The HRQoL of the patients was assessed with the EORTC QLQ-C30 module which has been developed and validated explicitly for patients diagnosed with cancer, in addition to the module BR23 which addresses patients with breast cancer and the module PR25 which addressed patients with prostate cancer. The QLQ-C30 is a 30-item general questionnaire that assesses a wide range of functional outcomes and symptoms relevant among oncology patients. It consists of five functional domains assessing physical role, cognitive, emotional, and social aspects, one global QOL domain, three symptom domains, five single items assessing other symptoms, and one item assessing financial impact. Each question/item was scored on a numeric scale from 1 to 4 (1 = “not at all”; 2 “a little”;3 “quite a bit”; 4 “very much”). The only exception was with last two items assessing overall health and overall QOL, both of which were scored from 1 (very poor) to 7 (excellent) [31].

The BR23 module consists of 23 items and covers symptoms as well as side effects related to different treatment modalities, body image, sexuality, and future perspective. The assessment comprises 5 domains: body image, sexuality, group symptoms, breast symptoms, and systemic therapy side effects. The subscale measuring body image includes only 4 items and does not measure a multidimensional construct of body image [32].

The PR 25 module consists of 25 items and measures symptoms and problems specifically orientated towards prostate cancer patients. It is composed to form subscales on urinary symptoms, bowel symptoms, hormone treatment related problems and sexual function [33].

### Statistical analyses

Chi-square tests (χ2), independent T-test, Paired T-test and Linear Mixed Models (LMM) were used to analyse the data. Categorical data were analysed using the chi-squared test. LMM was used to test differences within time between intervention and control groups, as we wanted to control for intra-subject correlation of response measurements [9]. Our initial fit for the LMMs was with the Unstructured Covariance structure since this often offers the best fit and requires no assumption in the error structure [34]. We additionally tested the Toeplitz structure and/or its special case of the AR autoregressive where it assumes that measurements that are next to each other are highly correlated and become less correlated as they become farther and farther apart [35]. In case where two covariance structures provided solutions, we utilised the Akaike Information Criteria (AIC) to select the best model fit. The smaller the AIC the better the model fit [36, 37]. LMM models were adjusted with moderate (or higher) correlated continuous measurements according the instruction by [38]. No adjustments were made for the demographic characteristics as no differences were observed at baseline and it is suggested that no more than a few covariates should be included in the analysis to reduce model complexity [38]. A maximum of p value = 0.10 was considered for significant baseline differences. A significant interaction term of condition (coded as 1 = Intervention, 0 = Control) by Time (pre-post) suggests that the change in outcomes over time is statistically different for the groups being compared The data collected for all EORTC QLQ C-30 items and BR23 and PR25 questionnaires were transformed to a 0–100 scale following EORTC guidelines. Unless otherwise stated, normally-distributed data are presented as mean and standard deviation (SD) and non-normally distributed data as median. Statistical significance overall, was taken at the two-sided 5% level (P <0.05). Analyses were done with the IBM 21 SPSS software.

## Results

### Characteristics of the sample

Data was collected over an 18-month recruitment process from 208 patients (104 male and 104 female). A percentage of 83% of the prostate cancer patients were diagnosed with stage T3a, Gleason score 8, and the remaining 17% with stage T3b, Gleason score 9. Patients with breast cancer were all diagnosed with clinical stage T3N1M0 Geographically all regions in Cyprus were represented in the sample. The majority of the participants belonged to the 51–60 and 41–50 age groups. Participants’ demographic variables were generally well-matched at baseline (Table 1). No significant differences with regards to Age (p = 0.351), Education (0.481), Diagnoses (p = 0.584) and Type of treatment (Breast cancer p = 0.533, Prostate p = 0.8568) (Table 1).

**Table 1:** Participant characteristics.

		Intervention group (n = 104)		Control group (N = 104)			
		N	%	N	%	X^2^	p
Residence						4,636	0,326
	Nicosia	42	40,4%	35	33,7%		
	Limassol	16	15,4%	17	16,3%		
	Paphos	30	28,8%	24	23,1%		
	Larnaka	14	13,5%	25	24,0%		
	Ammochostos	2	1,9%	3	2,9%		
Gender						—	—
	Male	52	50,0%	52	50,0%		
	Female	52	50,0%	52	50,0%		
Age						3,273	0,351
	31–40	7	6,7%	14	13,5%		
	41–50	30	28,8%	25	24,0%		
	51–60	43	41,3%	38	36,5%		
	>60	24	23,1%	27	26,0%		
Diagnoses						1,075	0,584
	T3a, Gleason score 8	41	39,4%	45	43,3%		
	T3b, Gleason score 9	11	10,6%	7	6,7%		
	T3N1M0 (Breast Cancer)	52	50,0%	52	50,0%		
Type of Cancer						—	—
	Breast cancer	52	50,0%	52	50,0%		
	Prostate cancer	52	50,0%	52	50,0%		
**Type of treatment**			0,0%		0,0%		
Breast Cancer						0,388	0,533
	Surgery, Adjuvant chemotherapya	16	30,8%	19	36,5%		
	Surgery, Adjuvant chemotherapya, radiation	36	69,2%	33	63,5%		
Prostate Cancer						0,769	0,8568
	Surgery (Radical Prostatectomy)	2	3,8%	2	3,8%		
	Surgery, Radiotherapy	7	13,5%	5	9,6%		
	Radiation, androgen deprivation therapy (ADT)	11	21,2%	9	17,3%		
	Androgen deprivation therapy (ADT), Adjuvant chemotherapyb	32	61,5%	36	69,2%		
Level of education						3,475	0,481
	No formal education	10	9,6%	14	13,5%		
	Primary school	16	15,4%	9	8,7%		
	Secondary school	25	24,0%	30	28,8%		
	Higher education (college/polytechnic)	27	26,0%	23	22,1%		
	University degree	26	25,0%	28	26,9%		

a Doxorubicin 60 mg/m 2 IV plus cyclophosphamide 600 mg/m 2 IV on day 1 every 21 days for four cycles followed by Docetaxel 100mg/m2 IV. Repeat cycle every 21 days for 4 cycles.

b Docetaxel dosed at 75 mg/m2 every 3 weeks + Prednisone 10mg.

### Pain

**Fig 2:**
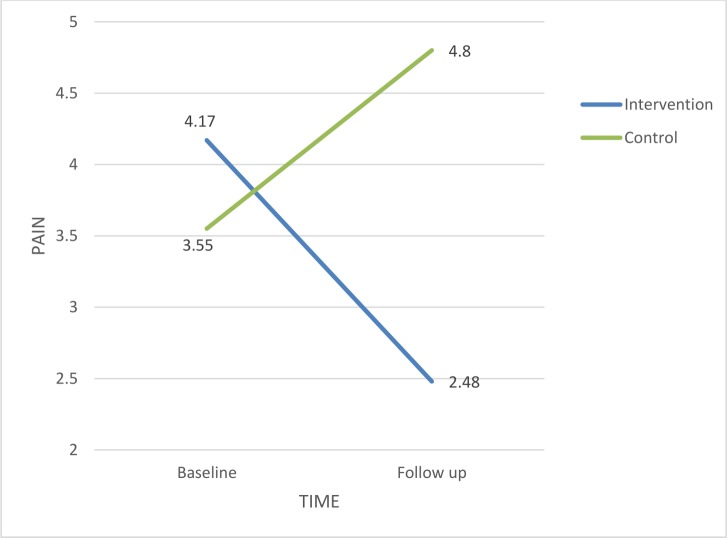
Estimated marginal means for Pain.

Patients in the intervention and control groups reported average pain levels at baseline (mean 4.17, SD 1. 47 and 3.55, SD 1. 73 respectively). No significant differences were found between prostate and breast cancer patients. Following the intervention, patients in the intervention group reported lower pain levels (mean 2.48, SD 1. 35) compared to those in the control group that experienced increased pain levels (mean 4.80, SD 1. 46). The intervention was statistically significant within time (F = 29.64, p<0.0001) (Table 2). Pain in the intervention group has declined and increased in the control group (Fig 2).

**Table 2:** Linear Mixed Models of Depression, Fatigue, Anxiety and Vomiting for the effect of intervention group. Adjusted for covariates.

	Dependent variable:							
	Depression (BDI-II)		Fatigue (CFS)		Anxiety (SAS)		Vomiting (INVR)	
Source	F	P	F	p	F	p	F	p
Intercept	5,30	0,022	16,52	<0,0001	168,41	<0,0001	118,92	<0,0001
Intervention Group	0,78	0,379	4,80	0,0296	1,08	0,2995	16,45	0,0001
Time (Pre-Post)	12,46	0,001	0,32	0,5696	0,35	0,5555	0,79	0,3752
Intervention Group*Time	**79,95**	**<0,0001**	**5,25**	**0,0225**	**7,22**	**0,0075**	**58,50**	**<0,0001**
Vomiting (INVR)	0,21	0,647	2,08	0,1504	0,40	0,5252		
Depression (BDI-II)			13,41	0,0003	111,36	<0,0001	0,27	0,6049
Fatigue (CFS)	13,61	0,0002			123,59	<0,0001	1,76	0,1851
Anxiety (SAS)	99,70	<0,0001	101,63	<0,0001			0,92	0,3390

Linear Mixed Models, Covariance Structure: Unstructured

Restricted Maximum Likelihood Estimation (REML)

Coding: Intervention group = 1, Control group = 0

### Fatigue

The control group’s mean total fatigue score on the CFS was 28.7 (SD 4.1). The mean score for each subscale was as follows: physical 15.2 (SD 7.1), affective 7.4 (SD 4.3), and cognitive 6.1 (SD 3.7). The intervention group’s mean total fatigue score was 27.6 (SD 3.9). Prostate cancer patients reported higher levels of fatigue compared to breast cancer patients (mean score 28.3 Vs mean score 24.1 respectively). The patients’ mean total fatigue score on the CFS following the intervention was 19.3 (SD 3.5) indicating a reduction in the perceived fatigue in this group of patients. After adjusting for correlated symptoms (Table 2), the LMM analysis revealed a significant condition by time interaction (F = 5.25, p = 0.0225).

### Nausea, vomiting and retching (INVR)

The mean distress level caused by nausea, vomiting and retching in the control group at baseline was 17.8(6.5) and increased to 22.7(5.3) at follow-up. In the intervention group at baseline the mean distress level was 25.4(5.9) while it decreased to 20.6(5.6) at follow-up. After adjusting for correlated symptoms (Table 2), the LMM analysis revealed a significant condition by time interaction time (F = 58.50, p<0.0001). Moreover, Nausea and Vomiting according to the EORTC QLQ-C30 reduced significantly in the Intervention group (t = -8.08, p<0.0001) and increased significantly in the controlled group (t = 8.63, p<0.0001) (Table 3). In terms of the experienced nausea, vomiting and retching, no differences were found between prostate and breast cancer patients.

**Table 3:** Mean Baseline and Change From Baseline (intervention) QLQ-C30 Scores.

Subscales	Baseline (pre intervention)		Follow Up (post intervention)			
	Intervention Group	Control Group	Intervention Group (Mean Change–p^†^)		Control Group (Mean Change–p^†^)	
Global QOL*	**54.9 (22.7)**	**51.9 (22.3)**	**+9.5**	**<0.0001**	**-10.7**	**<0.0001**
Functional Scales*						
Physical Functioning*	**66.5 (21.4)**	**68.1 (23.2)**	**+8.7**	**<0.0001**	**-3.4**	**<0.0001**
**Role functioning**	**69.2 (22.8)**	**71.1 (29.5)**	**+2.1**	**<0.0001**	**-1.5**	**<0.0001**
**Emotional functioning**	**59.8 (22.1)**	**63.3 (23.8)**	**+5.6**	**<0.0001**	**-4.2**	**<0.0001**
**Cognitive functioning**	**83.2 (20.1)**	**81.1 (20.3)**	**+0.3**	**0.009**	**-0.2**	**0.177**
**Social functioning**	**76.4 (25.8)**	**79.4 (24.5)**	**+1.0**	**0.036**	**-1.7**	**<0.0001**
Symptom Scales**						
**Fatigue**	**67.8 (19.6)**	**73.1 (21.8)**	**-17.1**	**<0.0001**	**+7.6**	**<0.0001**
**Nausea and Vomiting**	**34.5 (25.3)**	**37.7 (24.5)**	**-8.4**	**<0.0001**	**+2.2**	**<0.0001**
**Pain**	**45.9 (26.1)**	**44.9 (28.3)**	**-11.3**	**<0.0001**	**-1.0**	**0.0004**
**Dyspnea**	**28.2 (27.1)**	**29.2 (27.5)**	**+0.6**	**0.0044**	**+0.4**	**0.00034**
**Insomnia**	**21.2 (27.0)**	**19.6 (26.2)**	**-1.3**	**<0.0001**	**+0.9**	**<0.0001**
**Appetite loss**	**21.4 (29.3)**	**20.9 (27.8)**	**-1.0**	**0.00065**	**+3.3**	**<0.0001**
**Constipation**	**20.7 (23.6)**	**21.6 (24.4)**	**+0.8**	**<0.0001**	**+1.0**	**<0.0001**
**Diarrhea**	**15.5 (25.7)**	**14.7 (24.3)**	**-1.5**	**<0.0001**	**-0.3**	**<0.0001**
**Financial difficulties**	**32.1 (28.2)**	**32.0 (28.1)**	**-2.4**	**<0.0001**	**-2.0**	**<0.0001**

*Higher scores indicate better global Quality of Life and functioning

**Higher scores indicate worse symptoms

† Paired t-test

### Anxiety

Mean SAS score for the patients in the control group was 49.8 (SD = 8.9), ranging from 29 to 75/80 with breast cancer patients experiencing higher levels of anxiety compared to prostate cancer patients. The intervention group had a mean score of 39.2 (SD = 3.5), range = 21 to 66/80 (Table 4). When classified according to Zung’s [29] cut-off point (36 or above) for the presence of clinically significant anxiety, although the participants in the intervention group reported relatively high levels of anxiety these were found significantly lower than those in the control group. Explicitly only 25% of the participants in the intervention group scored above the cut-off point (after the intervention) compared to 78% in the control group. After adjusting for correlated symptoms (Table 2), the LMM analysis revealed a significant condition by time interaction for Anxiety (F = 7.22, p = 0.0075).

**Table 4:** BDI-II and SAS scores at baseline and post-intervention in the intervention and control groups.

	Intervention group				Control group			
	Baseline	Follow up	Δ	p^†^	Baseline	Follow up	Δ*	p^†^
BDI-II	27.3(7.6)	19.6(8.6)	7.7(6.9)	<0.0001	28.2(9.4)	35.2(12.0)	-7.0(7.8)	<0.0001
SAS	45.9(4.2)	39.2(3.5)	6.7(7.9)	<0.0001	43.5(8.6)	49.8(8.9)	-6.3(6.3)	<0.0001

*Δ, change from the baseline score

^**†**^ Paired t-test

### Depression

Patients in the control group demonstrated significantly higher BDI-II total mean scores compared to patients in the intervention group with breast cancer patients being more depressed. Explicitly, following the intervention the BDI-II mean scores for the intervention group was 19.60 (SD = 8.64) and for the control group was 35.21 (SD = 12.18). When classified according to Beck et al [30] guidelines, the participants in the intervention group were experiencing moderate depression whilst those in the control group experienced severe depression. A total of 35 (33.6%) of participants in the intervention group were moderately depressed at baseline (BDI-II ≥20 [30]), in comparison to the 15 participants (14.4%) at the post intervention evaluation. Whereas in the control group, the number of moderately depressed participants increased by 42% throughout the study period (X^2^  =  5.93; p  =  0.02). When the two groups were compared before and after the intervention for their mean depression level (BDI-II), the LMM analysis revealed a significant condition by time interaction (F = 79.95, p<0.0001) (Table 2).

### Health related Quality of Life

Assessing the results before the intervention with the independent T-test showed that the two groups were similar in the functional and symptomatic scales of quality of life. There was no statistically significant difference between the two groups before the intervention. Table 3 shows patients' functioning and global quality of life as measured by the EORTC QLQ-C30. Mean global QoL score changed significantly over time within and between groups. Explicitly, global quality of life scores showed an increase of 9.5 points in the intervention group compared to a decrease of 10.7 points in the control group (p<0.0001). This finding shows that the patients in the intervention group reported better HRQoL compared to those in the control group.

Changes of>10 points on the GQOL/QOL scale of the QLQ-C30 are considered meaningful in longitudinal studies [38]. With the exception of the cognitive functional scale patients in the intervention group reported higher (improved) levels of functioning compared to baseline measurements. Patients in the control group reported consistently lower levels of functioning with the emotional functioning demonstrating the highest decrease (-4.2 points). No changes were observed for the cognitive scale. After adjusting for correlated covariates, the LMM analysis revealed a significant condition by time interaction on the Global QoL (F = 29.64, p<0.0001) (Table 5).The mean QLQ-PR25 scores by assessment time are presented in Table 6. Scores on the EORTC-QLQ-PR25 decreased in the intervention group. Repeated measures showed significant and clinically relevant decreases after the intervention for the subscales of sexual functioning (P<0.001), and treatment-related functions (P<0.001). In the control group analyses showed increased scores for the subscales treatment-related symptoms and bowel symptoms. Also unchanged scores for the subscales incontinence aid, sexual interest and urinary symptoms. Only the sexual functioning subscale showed a 4-point increase in the control group.

**Table 5:** Linear Mixed Models of Global QoL and PAIN, for the effect of intervention group. Adjusted for covariates.

	Dependent variable:			
	Global QoL		PAIN Scale (1..10)	
Source	F	Sig.	F	Sig.
Intercept	546,95	<0,0001	0,34	<0,0001
Intervention group	17,05	<0,0001	16,47	<0,0001
Time (Pre-Post)	1,35	0,2459	0,18	0,6750
Intervention group * Time	**29,64**	**<0,0001**	**13,55**	**0,0003**
Vomiting (INVR)	2,25	0,1341	0,38	0,5384
Anxiety (SAS)	3,82	0,0514	14,56	0,0002
Depression (BDI-II)	11,76	0,0006	10,73	0,0012
Fatigue (CFS)	30,94	<0,0001	20,59	<0,0001

Covariance Structure: Unstructured

Restricted Maximum Likelihood Estimation (REML)

Coding: Intervention group = 1, Control group = 0

**Table 6:** Mean QLQ-PR25 Scores at Baseline and after the Intervention.

Subscales	Baseline (pre intervention)			Follow Up (post intervention)		
	Intervention Group	Control Group	p^†^	InterventionGroup	Control Group	p^†^
**Unirary Symptoms**	**72 (20.1)**	**55 (17.3)**	**<0.0001**	**61 (22.1)**	**56 (17.0)**	**0.0704**
Incontinence aid*	**80 (15.0)**	**72 (23.0)**	**0.0037**	**78 (19.1)**	**75 (20.0)**	**0.271**
**Bowel symptoms**	**36 (24.0)**	**35 (21.2)**	**0.751**	**35 (21.2)**	**46 (20.7)**	**0.0003**
**Treatment-related symptoms**	**78 (19.1)**	**81 (15.0)**	**0.211**	**67 (21.9)**	**92 (11.4)**	**<0.0001**
**Sexual interest**	**94 (9.2)**	**90 (13.4)**	**0.0137**	**82 (15.0)**	**90 (13.4)**	**0.0009**
Sexual functioning**	**90 (13.4)**	**95 (8.2)**	**0.00159**	**78 (19.2)**	**91 (9.8)**	**<0.0001**

* Patients who wear incontinence aids only

** Patients who were sexually active only

† independent samples t-test

Breast cancer patients' HRQoL scores as measured by the EORTC QLQ-BR23 are shown in Table 7. There were significant increases in the intervention group in all the functioning scores compared to the baseline assessment. Significant deteriorations were observed in the control group in relation to the body image subscale. Repeated measures showed significant and clinically relevant decreases for the subscales in group symptoms, systematic therapy side effects and dismay by hair loss in the intervention group following the intervention (P<0.001). The subscale, “upset by hair loss” showed a remarkable 21.3 points decrease in the intervention group.

**Table 7:** Mean QLQ-BR23 Scores at Baseline and Average Change by Intervention.

Subscales	Baseline (pre intervention)			Follow Up (post intervention)		
	Intervention Group	Control Group	p^†^	InterventionGroup	Control Group	p^†^
Functioning*						
**Body image**	**61.2 (33.4)**	**63.7 (35.1)**	**0.599**	**86.2 (17.8)**	**54.3 (24.2)**	**<0.0001**
**Sexual functioning**	**67.7 (26.3)**	**65.5 (30.2)**	**0.576**	**82.3 (22.2)**	**66.0 (31.1)**	**<0.0001**
**Sexual enjoyment**	**23.5 (28.7)**	**24.2 (20.5)**	**0.840**	**51.6 (25.3)**	**22.3 (28.5)**	**<0.0001**
**Future perspective**	**29.8 (17.2)**	**32.2 (16.9)**	**0.313**	**36.0 (25.6)**	**31.1 (16.7)**	**0.105**
Symptoms**						
**Arm symptoms**	**24.8 (19.9)**	**25.4 (21.1)**	**0.833**	**18.7 (12.2)**	**31.4 (28.7)**	**<0.0001**
**Breast symptoms**	**22.3 (14.9)**	**24.7 (20.0)**	**0.329**	**35.3 (27.9)**	**25.6 (19.2)**	**0.0043**
**Systematic therapy side effects**	**45.5 (25.0)**	**46.7 (21.0)**	**0.709**	**29.1 (17.5)**	**46.0 (21.2)**	**<0.0001**
**Upset by hair loss**	**38.9 (38.5)**	**37.0 (27.3)**	**0.682**	**17.6 (15.3)**	**30.0 (17.0)**	**<0.0001**

* Higher scores indicate better functioning

** Higher scores indicate worse symptoms

† Independent samples t-test

## Discussion

This was a randomised control trial designed to provide rigorous evidence on the effectiveness of Guided Imagery and Progressive Muscle Relaxation in patients diagnosed with either prostate or breast cancer and experience a cluster of symptoms. As these patients often receive chemotherapy as part of their treatment they experience numerous symptoms that can depilate their perceived quality of life. Despite the wide availability of studies exploring the effectiveness of CAM methods in a variety of treatment-related or cancer-related symptoms, there are no studies, in the extent of our knowledge that emphasise on these methods’ effectiveness when patients suffer from a cluster of symptoms. The uniqueness of this study makes it impossible for any comparisons with preceding studies to take place. Therefore any comparisons presented are drawn on the principle that these interventional methods targeted a single symptom in the preceding studies.

The GI and PMR intervention demonstrated strong evidence on their effectiveness in the management of symptom clusters in prostate and breast cancer patients. The findings showed that the intervention was significantly more effective in improving pain outcomes in the intervention group compared to the control. These findings coincide with those of earlier studies suggesting a positive effect in pain management, in patients diagnosed with cancer [39, 40]. Bardia et al [41] in a systematic review of RCT testing, the effectiveness of various CAM interventions for cancer-related pain concluded that imagery alone or in combination with relaxation techniques can be effective in reducing pain. From the patients’ point of view, Kwekkeboom et al [14] in a qualitative study reported that the majority of participants perceived that these interventions worked for their pain and, in fact, many reported a clinically significant change in pain with the interventions.

The data analyses revealed statistically significant reduction of perceived fatigue following the intervention. The reduction was reflected on the physical, affective, and cognitive subscales of the Cancer Fatigue Scale. Kim and Kim [42] in a small, randomized, pilot trial evaluated a relaxation/breathing exercise intervention compared with a control group for fatigue experienced by patients getting a stem cell transplant. The researchers found statistically significant lower levels of fatigue in the group of patients receiving the breathing intervention compared to the control group. Gaston-Johansson et al [43] in a randomised control trial with 110 patients found that a comprehensive coping strategy program was effective in significantly reducing nausea and fatigue in patients with breast cancer who underwent autologous bone marrow/peripheral blood stem cell transplantation.

Findings from the present study provided evidence that chemotherapy-related nausea, vomiting and retching experience, occurrence and distress were significantly lower in the intervention group compared to the control group. Morrow and Hickok [44] reported that progressive muscle relaxation training was effective in preventing as well as decreasing the frequency of post-chemotherapy nausea and vomiting. In a study of 60 women with breast cancer, Yoo et al [18] assessed the effectiveness of progressive muscle relaxation training and guided imagery in reducing the anticipatory nausea and vomiting, and post-chemotherapy nausea and vomiting in patients with breast cancer. Patients in the intervention group experienced reduced nausea and vomiting before and after chemotherapy. Results from a study by Arakawa [45] in 60 Japanese patients who were hospitalized in a cancer centre receiving chemotherapy verified the effectiveness of progressive muscle relaxation in reducing total scores used to measure nausea, vomiting, and retching. Scott et al [46] compared a clinical relaxation program that included guided imagery with a standard antiemetic drug protocol for chemotherapy. The patients in the relaxation program experienced less and shorter episodes of nausea and vomiting. Troesch et al [47] tested the effectiveness of guided imagery in decreasing nausea, vomiting, and retching occurrence and distress in a convenience sample of 28 patients receiving cisplatin-based chemotherapy. Findings revealed no statistically significant difference in this measurement between the two groups however the guided imagery group expressed a significantly more positive experience with chemotherapy (p = 0.0001).

GI and PMR were found effective in decreasing anxiety and depression in prostate and breast cancer patients in this study but also in preceding ones. Their effectiveness was also demonstrated by the saliva a-amylase and saliva cortisol biomarkers response to the intervention that were also recorded in this study but reported elsewhere [48]. León-Pizarro et al [49] in a randomized study aimed to determine the efficacy of psychological intervention consisting of PMR and GI to reduce anxiety and depression in gynaecologic and breast cancer patients undergoing brachytherapy during hospitalization. Sixty-six patients were included in the study with only those in the intervention group (n = 32) receiving training in relaxation and guided imagery. The study group demonstrated a statistically significant reduction in anxiety (p = 0.008) and depression (p = 0.03) compared to the control group. Sloman [50] in an Australian community-based nursing study compared the effects of progressive muscle relaxation and guided imagery on anxiety, depression, and quality of life in 56 patients with advanced cancer. Patients were randomly assigned to 1 of 4 treatment conditions: (a) progressive muscle relaxation training, (b) guided imagery training, (c) both of these treatments, and (d) control group. There was no significant improvement for anxiety; however, significant positive changes occurred for depression and QoL.

In the study by Yoo et al [18] that assessed the effectiveness of PMR training and GI in breast cancer patients, the patients in the intervention group were found significantly less anxious and depressive. As a result of the reduction in chemotherapy side effects, the patients also reported higher levels of QoL. The effectiveness of GI in alleviating mood disturbance and improving QoL was studied in a group of 8 cancer patients [51]. Patients were assigned to either an intervention or a wait-list control group. Individuals who participated in the guided imagery sessions scored better on both mood scores and QoL scores at post-test than those participating in the control group. Additionally, mood and quality of life scores continued to improve in the intervention group, even after sessions were completed. These findings coincide with those reported in the current study where the patients in the intervention group experienced significantly less severe symptoms an aspect that appeared to have an impact on their overall perceived HRQoL. The patients in this group reported better scores, hence better functioning than those in the control group. The debilating effect of the multiple symptoms was evident in the lower HRQoL reported by the control group, with the patients reporting consistently lower levels of functioning with the emotional functioning demonstrating the higher decrease. The cancer specific HRQoL subscales (PR25 and BR23) coincided with the results found for the generic HRQoL subscale (QLQ-C30).

The main limitation of the study was the inability to perform a double blind randomized control trial, as patients were difficult to be masked based on the intervention provided. Therefore the research team acknowledges the difficulty to control the placebo effect. Although patients reported that they adhered to the daily session of GI + PMR, the researchers are not able to know whether the patients performed the protocol in full each time. Furthermore, the researchers are not aware if the patients during these unsupervised sessions chose an external stimuli free environment or whether they had to interrupt the session. As any interruptions in the protocol might have impacted on its effectiveness, the researchers acknowledge that some of these cases have likely been included in the study. Despite the limitations of the trial, the rather rigorous design and implementation allow for the generalizability of the findings in these group of patients.

## Conclusion

In conclusion, this randomised control trial despite its limitations, provided evidence that supported the integration of GI and PMR as part of the comprehensive symptom management of the multiple symptoms experienced during chemotherapy, by patients diagnosed with prostate and breast cancer. However, any future studies in this field should adopt a double blind design and emphasize on the longer effects of these methods in order to provide stronger evidence for their effectiveness in the symptom clusters management.

## Supporting Information

S1 File Related manuscript. (PDF)

S2 File Protocol in English. (PDF)

S3 File Protocol in Greek. (PDF)

S4 File CONSORT checklist. (DOC)
