# Supplementary material for: Guided Imagery And Progressive Muscle Relaxation as a Cluster of Symptoms Management Intervention in Patients Receiving Chemotherapy: A Randomized Control Trial
Source: PLoS One. 2016 Jun 24;11(6):e0156911. doi: 10.1371/journal.pone.0156911 (PMC4920431; doi:10.1371/journal.pone.0156911)
Supplement: S2 File — (PDF) [file pone.0156911.s002.pdf]

Research Protocol Submitted To The Cyprus National Bioethics Committee for Evaluation

<u>Research Title</u>

«THE USE OF GUIDED IMAGERY AND PROGRESSIVE MUSCLE RELAXATION FOR THE BETTER MANAGEMENT OF SYMPTOMS EXPERIENCED BY CANCER PATIENTS AND FOR IMPROVING QUALITY OF LIFE»

<u>Research Details</u>

<u>Introduction</u>

1. **Design:** Randomized Controlled Trial

2. **Number of Centers and Organizations Participating:** 1 – Cyprus University of Technology, Pancyprian Association for Cancer Patients and Friends, Anticancer Society, Bank of Cyprus Oncology Centre, Oncology Centers of Public General Hospitals

3. **Total number of person that will take part in the study: 6**

4. **Rationale for the study:** This trial aims to explore the effectiveness of cognitive-behavioral and relaxations techniques, specifically Guided Imagery and Progressive Muscle Relaxation in improving quality of life (Quality of Life, EORTC QLQ-C30 (Breast Module-BR23 and Prostate Module-PR25), in decreasing the experienced pain levels (pain intensity, numeric -pain intensity-rating scale -NRS), in reducing fatigue (Cancer Fatigue Scale - CFS), in reducing nausea, vomiting and retching (Revised Rhodes index of nausea, vomiting and retching-INVR), in reducing depression, (depression - Beck Depression Inventory) and in reducing anxiety (psychometric and biological measurements of anxiety - Self-Rating Anxiety Scale, SAS + SALIVARY CORTISOL – SALIVARY α-AMYLASE)

5. **Background:** Cognitive-Behavioural and relaxation techniques (often referred as Complementary and Alternative Therapies or Mind-Body Therapies) including Guided Imagery and Progressive Muscle Relaxation are often recommended as complementary strategies for maximizing the pain relief in patients with cancer. Pain that is unresponsive to pharmacological means, is a frequent cause for admission in the hospital mainly for patients who receive complicated cancer treatment regimens such as in the cases of BMT and for patients with advanced cancer.

   Statistics show that about 79% of patients with cancer and receive treatment experience at some point pain. The primary management of cancer pain is focused on the use of analgesic drugs and other adjuvant drugs. Whilst complementary

therapies are offered in some clinical settings, it is believed that they remain largely underutilized.

Cognitive-Behavioral and relaxation techniques (often referred as Complementary and Alternative Therapies or Mind-Body Therapies) including Guided Imagery and Progressive Muscle Relaxation are also recommended for promoting the perception of pain control (self-management). Guided Imagery utilizes ones imagination to create mental images that have the potential to distract the attention of the person away from pain and even altered the perception of pain. For example, patients can visualize themselves being on a beach or a mountain as stress reducing sceneries and forger about their pain. They can also visualize pain and altered this image so that they altered the experience of pain.

Systematic reviews on cognitive-Behavioral and relaxation techniques have shown they can be effective in reducing cancer pain. However, the researchers reported that the techniques used by the patients varied. Donovan και Laack in a study with 163 patients suffering from chronic cancer pain explored the effectiveness of various complementary and alternative techniques in managing the pain. They concluded that none of the techniques was effective for all the patients in the study. Relaxation techniques were found effective for pain management for some patients but had no effect on others. Kwekkeboom et al utilized Guided Imagery as a pain relief strategy for hospitalized patients experiencing cancer pain. The majority of the patients reported lower levels of

pain whilst 10% reported that they did not experience any changes in their pain levels. Other studies also highlight the potential benefit of cognitive-Behavioral and relaxation techniques in other symptoms such as fatigue, nausea and vomiting, anxiety and depression. None however of the preceding studies explored their benefit when these symptoms occur simultaneously.

6. **Assumptions made by the study.** The basic assumption is that the tested techniques will have a positive impact on the patients in the experimental group. This positive impact will include:
    - Improved of quality of life,
    - Decrease in pain levels,
    - Decrease in fatigue levels
    - Decrease in nausea and vomiting levels,
    - Decrease in depression levels
    - Decrease in anxiety levels.

7. **Aim of the study:** To explore the effectiveness of Guided Imagery and Progressive Muscle Relaxation in managing pain, fatigue, nausea and vomiting, in decreasing anxiety and depression and improving the quality of life of cancer patients.

8. **Objective Targets of the study:** Evaluating the effectiveness of these techniques in managing multiple symptoms and as a result improving the quality of life.

9. **Benefit from the study (In Cyprus and abroad):** Given that positive results will arise from the study a confident recommendation of utilizing these cognitive-behavioral and relaxation techniques in the care plan of the patient with cancer can then be made. Training patients and their informal caregivers on these techniques so that they can independently implement these. Improving the patients' quality of life and promoting the sense of well-being. Decreasing admission in oncology units due to the limited or lack of control of these symptoms (e.g. pain). Promoting patients' sense of control (and for their informal caregivers).

10. **Study design: See Diagram 1** (Appendix)

11. **Sample size (estimation): 200 (100+100)**

12. **Sample size calculation:** Type I error, power, assumptions on response rate and standard deviation, and expected treatment effect were taken assumptions were taken into consideration in calculating sample size. The type I error and power were set at conventional levels (5% for type I error and 80% for power). Assumptions were based on preceding studies' available data and published results. Based on these, a 25% will be added in the final calculated sample to ensure the size was large enough to detect any effect after possible low response rates, drop-outs, refusals and losses in follow-ups.

13. **Selection criteria:** Eligible participants need to be adults (18 or above), with a clinical diagnosis of prostate or breast cancer at least 6 months prior their admission to the study and are willing to be included in the study. Eligible patients are those that at any stage of the disease have tried one or both of the tested techniques. Eligible participants will need to be able to speak and write Greek.

14. **Exclusion criteria:** Patients were excluded from the study if they were newly diagnosed (< 6 months) or were at the end-of-life stage. Patients were also excluded if they visual and/or hearing impairment and/or cognitive impairment, xerostomia and/or oral mucositis.

15. **Procedures:** Patient assessment for eligibility and recruiting will take place at the out-patient clinics of the participating centers. Eligible participants, following their informing session will be called to sign an informed consent. Those that will be included in the study will have 3 supervised sessions of Guided imagery and Progressive muscle relaxation. Patients will be called to complete concise questionnaires before the intervention and at the end of all of the intervention sessions. Furthermore, prior of the intervention and following each supervised session a saliva sample will be collected.

16. **Research Tools: The various symptoms and quality of life will be assessed with the following tools:** EORTC QLQ-C30 (Breast Module-BR23 and Prostate Module-PR25), pain intensity- (NRS), Beck Depression Inventory II, Self-Rating Anxiety Scale (SAS), Revised Rhodes index of nausea, vomiting and retching-INVR and interviews. Saliva samples will be assessed for SALIVARY CORTISOL, SALIVARY α-AMYLASE.

17. **Στατιστική ανάλυση:** Statistical analyses will be performed by a statistician and it will include descriptive and inferential statistics. Descriptive statistics will include charts with means and standard deviations. Inferential statistics will include ANOVA, and paired t tests calculations. Qualitative data will be analyzed with content analysis.

18. **Informed consent to the study:** Participants will sign a consent form following the receipt of oral and written information on the study. Participants will also be informed on their rights by taking part in this study, and especially their right to leave the study at any time without questions asked.

19. **Responsible for the study is the PI**

20. N/A (reimbursement of the patients taking part in the study)

21. N/A (use of genetic data)

22. No personal data will accompany the publications. Only general demographic data will be presented.

23. Demographic data will include residence, gender, age, time from diagnosis and cancer type.

24. No other personal data will be disseminated – N/A

25. No genetic data will be disseminated – N/A

26. No genetic or other biological data will be disseminated – N/A – Following saliva analyses, all samples will be destroyed.

27. Access to information from the study's participants: Results will be available to all the participants as well as first degree relatives.

28. Time for storing and destroying samples and data: Data and samples will be saved for the minimal time possible as to allow publications of the results. Biological samples will be destroyed immediately following their analyses based on protocol for the destruction of biological samples.

29. Complaints: Any complaints or professional misconduct by members of the research team can be submitted in the PI. Furthermore, these can also be addressed to Dr Charalambous Chrysostomo, Chief of the Research and Internal Relations of the Cyprus University of Technology:

Cyprus University of Technology

Athenwn 80, TT 3036 Limassol

Tel 00357 25002562 Fax 0035725002763

Email. c.chrisostomou@cut.ac.cy

Selected References

Eisenberg DM, Davis RB, Ettner SL, et al. Trends in alternative medicine use in the United States, 1990–97: results of a follow-up national survey. JAMA. 1998;280:1569–75.

Wootton JC, Sparber A. Surveys of complementary and alternative medicine: part I. General trends and demographic groups. J Altern Complement Med. 2001;7:195–208.

Kiecolt-Glaser JK, Mcguire L, Robles TF, Glaser R. Psychoneuroimmunology and psychosomatic medicine: back to the future. Psychosom Med. 2002;64:15–28.

Stefano GB, Fricchione GL, Slingsby BT, Benson H. The placebo effect and relaxation response: neural processes and their coupling to constitutive nitric oxide. Brain Res Rev. 2001;35:1–19.

Sobel DS. Mind matters, money matters: the cost-effectiveness of mind-body medicine. JAMA. 2000;284:1705.

Sobel DS. The cost-effectiveness of mind-body medicine interventions. Prog Brain Res. 2000;122:393–412.

Friedman R, Sobel D, Myers P, Caudill M, Benson H. Behavioral medicine, clinical health psychology, and cost offset. Health Psychol. 1995;14:509–18.

MacLennan AH, Wilson DH, Taylor AW: The escalating cost and prevalence of alternative medicine. Preventive Medicine 2002, 35:166-173.

Eisenberg DM, Davis RB, Ettner SL, Appel S, Wilkey S, Van Rompay M, Kessler RC: Trends in alternative medicine use in the United States, 1990-1997. JAMA 1998, 280:1569-1515.

White AR, Ernst E: Economic analysis of complementary medicine: a systematic review. Complementary Therapies in Medicine 2000, 8:111-118.

Pelletier KR, Astin JA: Integration and reimbursement of complementary and alternative medicine by managed care and insurance providers: 2000 update and cohort analysis. Alternative Therapies 2002, 8:38-48.

Neumann PJ: Evidence-based and value-based formulary guidelines. Health Affairs 2004, 23:124-134.

Sullivan SD, Lyles A, Luce B, Grigar J: AMCP guidance for submission of clinical and economic evaluation data to support formulary listing in US health plans and pharmacy

benefits management organizations. Journal of Managed Care Pharmacy 2001, 7:272-282.

Claxton K, Neumann PJ, Araki S, Weinstein MC: Bayesian value-of information analysis: an application to a policy model of Alzheimer's disease. International Journal of Technology Assessment in Health Care 2001, 17:38-55.

Claxton K, Posnett J: An economic approach to clinical trial design and research priority-setting. Medical Economics 1996, 5:513-524.

Παραρτήματα

Σχεδιασμός της έρευνας

Patients assessed for eligibility
Desired number for participation (minimum number N=200)

↓

Randomization of participants
N=200

Usual treatment + [GI + PMR]
100 participants allocated to receive guided imagery and PMR sessions

Measurements will be made before and after each trial (saliva amylase α and cortisol).
Participants will also rate pain intensity, N&V occurance, stress levels and quality of life

Usual treatment ONLY
100 participants allocated – did not receive guided imagery and PMR sessions

The same measurements will be made at the same periods as the intervention group

T
I
M
E

Post Study Interview

Data Analysis

Data Analysis

Table 1 Ερωτήσεις Συνέντευξης

**Τι πιστεύεται για τις τεχνικές του κατευθυνόμενου οραματισμού και της προοδευτικής μυϊκής χαλάρωσης ?**<br><br>**1. Το απολαύσατε?**<br><br>**2. Πιστεύετε ότι έχουν δουλέψει για εσάς?**<br>**α. Αν όχι: Έχετε κάποια ιδέα για τους λόγους που δεν δούλεψαν για σας?**<br>**β. Αν ναι: Έχετε κάποια ιδέα για τους λόγους που δούλεψαν για σας?**<br>**Πόσο κράτησε η επίδραση τους για σας?**<br><br>**3. Ήταν η διάρκεια τους ικανοποιητική? Θα προτιμούσατε μεγαλύτερη, μικρότερη, ή την ίδια?**<br><br>**4. Νιώθετε ότι δοκιμάζοντας τις τεχνικές 4 φορές κατω απο καθοδήγηση ήταν αρκετές για να διαπιστώσετε αν ήταν βοηθητικές για σας ή όχι?**
