# Supplementary material for: Guided Imagery And Progressive Muscle Relaxation as a Cluster of Symptoms Management Intervention in Patients Receiving Chemotherapy: A Randomized Control Trial
Source: PLoS One. 2016 Jun 24;11(6):e0156911. doi: 10.1371/journal.pone.0156911 (PMC4920431; doi:10.1371/journal.pone.0156911)
Supplement: S3 File — (PDF) [file pone.0156911.s003.pdf]

Πρωτόκολλο ερευνητικού έργου που υποβάλλεται για βιοηθική αξιολόγηση στην Εθνική Επιτροπή Βιοηθικής Κύπρου

Τίτλος ερευνητικού έργου

«ΕΦΑΡΜΟΓΗ ΤΟΥ ΚΑΤΕΥΘΥΝΟΜΕΝΟΥ ΟΡΑΜΑΤΙΣΜΟΥ ΚΑΙ ΤΗΣ ΠΡΟΟΔΕΥΤΙΚΗΣ ΜΥΙΚΗΣ ΧΑΛΑΡΩΣΗΣ ΓΙΑ ΤΗΝ ΒΕΛΤΙΩΣΗ ΤΗΣ ΔΙΑΧΕΙΡΙΣΗΣ ΣΥΜΠΤΩΜΑΤΩΝ ΚΑΙ ΤΗΣ ΠΟΙΟΤΗΤΑΣ ΖΩΗΣ ΣΕ ΑΤΟΜΑ ΜΕ ΚΑΡΚΙΝΟ»

Στοιχεία ερευνητικού έργου

Εισαγωγή

1. **Είδος προγράμματος:** Κλινική , τυχαιοποιημένη δοκιμασία ελέγχου

2. **Αριθμός φορέων που θα λάβουν μέρος στο πρόγραμμα:** 1 - Τεχνολογικό Πανεπιστήμιο Κύπρου, Παγκύπριος Σύνδεσμος Καρκινοπαθών και Φίλων, Αντικαρκινικός Σύνδεσμος, Ογκολογικό Κέντρο Τράπεζας Κύπρου, Ογκολογικά Κεντρα Γ. Νοσοκομείων)

3. **Συνολικός αριθμός των ατόμων που θα συμμετάσχουν στο πρόγραμμα:** 6

4. **Αιτιολόγηση της μελέτης:** Αυτή η τυχαιοποιημένη μελέτη στοχεύει στην διακρίβωση της αποτελεσματικότητας ψυχολογικών παρεμβάσεων που αποτελούνται από τον Κατευθυνόμενο Οραματισμό και την Προοδευτική Μυϊκή χαλάρωση στην βελτίωση της ποιότητας ζωής (Quality of Life, EORTC QLQ-C30 (Breast Module-BR23 and Prostate Module-PR25), στην μείωση του πόνου (pain intensity, numeric -pain intensity- rating scale -NRS), στην μείωση της κόπωσης (Cancer Fatigue Scale - CFS), στην μείωση της ναυτίας και έμετου (Revised Rhodes index of nausea, vomiting and retching-INVR), στην μείωση της κατάθλιψης (depression - Beck Depression Inventory) και στην μείωση του άγχους μέσω ψυχομετρικών και βιολογικών μετρήσεων (anxiety - Self-Rating Anxiety Scale, SAS + SALIVARY CORTISOL – SALIVARY α-AMYLASE

5. **Υπόβαθρο του προγράμματος:** Οι Γνωστικές-συμπεριφορικές στρατηγικές (συχνά αναφέρονται ως Εναλλακτικές και Συμπληρωματικές Θεραπείες ή Στρατηγικές Νου-Σώματος), συμπεριλαμβανομένου του Κατευθυνόμενου Οραματισμού και της Προοδευτικής Μυϊκής χαλάρωσης, συνιστώνται ως συμπληρωματικές στρατηγικές για να μεγιστοποιήσουν την ανακούφιση από τον πόνο του καρκίνου. Ο πόνος ο οποίος δεν μπορεί να ρυθμιστεί φαρμακευτικά αποτελεί μια συχνή αιτία για νοσηλεία, κυρίως για τους ασθενείς που υποβάλλονται σε πολύπλοκα σχήματα θεραπείας του καρκίνου, όπως η χημειοθεραπεία ή μεταμόσχευση μυελού των οστών, και για τους ασθενείς σε προχωρημένα στάδια της νόσου.

Οι εκτιμήσεις δείχνουν ότι το 79% των ασθενών με καρκίνο που νοσηλεύονται, βιώνουν την εμπειρία του πόνου. Η πρωταρχική αντιμετώπιση του πόνου που σχετίζεται με τον καρκίνο εστιάζεται στη χρήση των αναλγητικών και επικουρικών φάρμακων. Ενώ οι συμπληρωματικές θεραπείες μπορούν να προσφέρονται σε ορισμένους κλινικούς χώρους, εντούτοις θεωρούνται ότι κατά ένα μεγάλο ποσοστό παραμένουν ανεκμετάλλευτες.

Οι Γνωστικές-συμπεριφορικές στρατηγικές, συμπεριλαμβανομένου του Κατευθυνόμενου Οραματισμού και της Προοδευτικής Μυϊκής χαλάρωσης συνιστώνται ως ενισχυτικές παρεμβάσεις για την βελτίωση της ανακούφισης από τον πόνο και την προαγωγή της αντίληψης των ασθενών για τον έλεγχο του πόνου. Ο Κατευθυνόμενος Οραματισμός συνεπάγεται τη χρήση της φαντασίας για να δημιουργηθούν ψυχικές εικόνες οι οποίες έχουν την δυνατότητα να αποσπούν την προσοχή μακριά από τον πόνο ή να μεταβάλλουν την ίδια την αίσθηση του πόνου. Για παράδειγμα, οι ασθενείς μπορούν να φανταστούν ότι βρίσκονται σε μια παραλία ή σε μια βουνοπλαγιά για να διεγείρουν την χαλάρωση και να αποσπάσουν την προσοχή από τον πόνο. Μπορούν επίσης να φανταστούν τον πόνο και να χειραγωγήσουν αυτή την εικόνα ώστε να αλλάξουν την αίσθηση του πόνου.

Συστηματικές ανασκοπήσεις των γνωστικών-συμπεριφορικών στρατηγικών συμπεριλαμβανομένων του Κατευθυνόμενου Οραματισμού και της

Προοδευτικής Μυϊκής χαλάρωσης έχουν δείξει ότι είναι αποτελεσματικές στην μείωση του πόνου του καρκίνου. Ωστόσο, οι ερευνητές ανέφεραν διακύμανση όσον αφορά τις απαντήσεις των ερωτηθέντων σχετικά με τον τύπο γνωστικής-συμπεριφορικής στρατηγικής που εφάρμοσαν για αντιμετώπιση του πόνου. Οι Donovan και Laack μελέτησαν 163 ασθενείς με χρόνιο καρκινικό πόνο για τη χρήση διαφόρων μη φαρμακευτικών παρεμβάσεων αντιμετώπισης του πόνου και διαπίστωσαν ότι καμία από τις θεραπείες δεν ήταν αποτελεσματική για όλους τους ασθενείς. Για παράδειγμα, οι τεχνικές χαλάρωσης ήταν ιδιαίτερα αποτελεσματικές για ορισμένους ασθενείς, ενώ δεν είχαν καμία επίδραση στον πόνο για άλλους. Οι Kwekkeboom και συνεργάτες εφάρμοσαν την τεχνική του κατευθυνόμενου οραματισμού σε νοσοκομειακούς ασθενείς με καρκινικό πόνο και διαπίστωσαν ότι, ενώ η πλειοψηφία βίωσε κάποιο όφελος, το 10% ανέφεραν ότι δεν βιώσαν καμία αλλαγή στον πόνο τους. Αλλες μελέτες τονίζουν το πιθανό όφελος απο την χρήση των γνωστικών-συμπεριφορικών στρατηγικών και στρατηγικών χαλάρωσης και σε άλλα συμπτώματα όπως η κόπωση, ναυτια και έμετος, άγχος και κατάθλιψη. Καμία όμως από αυτές τις μελέτες δεν έχει διερευνήσει την αποτελεσματικότητα αυτών των στρατηγικών όταν τα συμπτώματα εμφανίζονται ταυτόχρονα.

6. **Υποθέσεις που θέτει το Πρόγραμμα** Η βασική υπόθεση που θέτει το πρόγραμμα είναι ότι οι γνωστικές-συμπεριφορικές στρατηγικές που θα χρησιμοποιηθούν ως μέρος του προγράμματος θα έχουν θετικό αντίκτυπο στους συμμετέχοντες (ομάδα παρέμβασης). Ο θετικός αυτός αντίκτυπος μεταφράζεται

- σε βελτίωση της ποιότητας ζωής,
- στην μείωση του πόνου,
- στην μείωση της κόπωσης
- στην μείωση της ναυτίας και του έμετου
- στην μείωση της κατάθλιψης και
- στην μείωση του άγχους.

7. **Σκοπός του Προγράμματος:** Να αξιολογηθεί η αξία των γνωστικών-συμπεριφορικών στρατηγικών στην διαχείριση του πόνου, της κόπωσης, της ναυτίας και έμετου, στην μείωση της κατάθλιψης και του άγχους και στην βελτίωση της ποιότητας ζωής των ασθενών με καρκίνο.

8. **Αντικειμενικοί στόχοι του Προγράμματος:** Αξιολόγηση της αποτελεσματικότητας του κατευθυνόμενου οραματισμού και της προοδευτικής μυϊκής χαλάρωσης στην διαχείριση πολλαπλών συμπτωμάτων και ως αποτέλεσμα στην βελτίωση της ποιότητας ζωής.

9. **Όφελος που θα προκύψει από το Πρόγραμμα:** Ενσωμάτωση των συγκεκριμένων γνωστικών-συμπεριφορικών στρατηγικών στο πρόγραμμα φροντίδας που παρέχεται στους ασθενείς με καρκίνο. Δυνατότητα εκπαίδευσης των ασθενών και των οικογενειακών φροντιστών τους για εφαρμογή των στρατηγικών αυτών από τους ιδίους μετά το εξιτήριο. Βελτίωση της ποιότητας ζωής των ασθενών και προαγωγή του αισθήματος ευεξίας. Περιορισμός των

εισαγωγών στις ογκολογικές μονάδες εξαιτίας της αδυναμίας ελέγχου κάποιων συμπτωμάτων (π.χ πόνος). Προαγωγή του αισθήματος ελέγχου που νιώθουν οι ασθενείς (και οι οικογενειακοί φροντιστές τους).

10. **Σχεδιασμός του προγράμματος:** Βλέπε σχήμα 1 (Παραρτήματα)

11. **Αριθμός του μεγέθους του δείγματος (αναμενόμενο): 200 (100+100)**

12. **Αιτιολόγηση για τον αριθμό του δείγματος:** Ο υπολογισμος του δειγματος εγινε με βαση το Στατιστικό λάθος τύπου Ι, την ισχύ, τις παραδοχές σχετικά με το ρυθμό ανταποκρισης και την τυπική απόκλιση καθώς και την αναμενόμενη αποτελεσματικότητα της παρέμβασης. Το Στατιστικό λάθος τύπου Ι και η ισχύς τέθηκαν στο 5% (for type I error) και 80% (for power). Οι παραδοχές βασιστηκαν σε προηγηθήσες μελέτες και σημοσιευμένα αποτελέσματα. Με βασή αυτά τα δεδομένα ενα ποσοστό 25% θα προστεθεί στο τελικό υπολογιζόμενο δείγμα ώστε να ληφθούν υπόψη όλες οι παραδοχές που αναφέρθηκαν προηγουμένως.

13. **Κριτήρια εισδοχής στο πρόγραμμα:** Για το πρόγραμμα ως υποψήφιοι συμμετέχοντες θα θεωρηθούν ενήλικες (18 ετών και άνω) οι οποίοι έχουν διαγνωσθεί με καρκίνο του προστάτη ή καρκίνο του μαστού τουλάχιστον πριν από 6 μήνες και επιθυμούν να συμμετάσχουν στην έρευνα. Οι υποψήφιοι συμμετέχοντες στο πρόγραμμα δεν θα πρέπει σε κανένα στάδιο της νόσου να έχουν δοκιμάσει μια (η και τις δυο) από τις γνωστικές-συμπεριφορικές

στρατηγικές που θα εφαρμοστούν ως μέρος του προγράμματος. Οι συμμετέχοντες θα πρέπει να μιλούν και να κατανοούν την ελληνική γλώσσα πολύ καλά.

14. **Κριτήρια για μη εισδοχή στο πρόγραμμα:** Η είσοδος των πασχόντων στη μελέτη πρέπει να γίνεται σε σημείο που να επιτρέπει την εφαρμογή της θεραπευτικής παρέμβασης για εύλογο χρονικό διάστημα. Επομένως, πρέπει να αποκλείονται από τη μελέτη οι πάσχοντες που βρίσκονται σε αρχικό (<6 μήνες) ή τελικό στάδιο της πάθησης, οπότε δεν επιτρέπεται η εφαρμογή της μελετώμενης θεραπευτικής παρέμβασης. Αποκλείονται ασθενείς με επηρεασμένες γνωστικές λειτουργίες, επηρεασμένο επίπεδο όρασης και ακοής και επηρεασμένο επίπεδο συνείδησης, με ξηροστομία και στοματοβλεννογονίτιδα.

15. **Διαδικασίες και μέθοδοι:** Η αναγνώριση και η παραπομπή των πιθανών υποψηφίων-ασθενών που πληρούν τα κριτήρια εισδοχής στο πρόγραμμα θα γίνει στα εξωτερικά ιατρεία των συμβαλλόμενων κέντρων φροντίδας. Μετά την ενημέρωση των υποψηφίων-ασθενών, αυτοί που ενδιαφέρονται για να συμμετάσχουν στο πρόγραμμα θα τους ζητηθεί να υπογράψουν την φόρμα συγκατάθεσης. Όσοι αποφασίσουν να συμμετάσχουν στο πρόγραμμα θα βιώσουν 3 συνεδρίες κατευθυνόμενου οραματισμού σε συνδυασμό με προοδευτική μυϊκή χαλάρωση. Θα τους ζητηθεί να συμπληρώσουν τα σχετικά ερωτηματολόγια πριν από την συνεδρία, ενώ μετά το τέλος της συνεδρίας θα τους ζητηθεί ένα δείγμα σάλιου το οποίο θα σταλεί για εργαστηριακό έλεγχο της κορτιζόλης και της α-

αμυλάσης. Μετά το πέρας και των 3 συνεδρίων θα τους ζητηθεί να συμπληρώσουν ξανά τα ίδια ερωτηματολόγια.

16. **Τρόποι επιμέτρησης ή εκτίμησης των αποτελεσμάτων:** Η αξιολόγηση των διαφόρων παραμέτρων θα γίνει με την χρήση σταθμισμένων ερωτηματολογίων (EORTC QLQ-C30 (Breast Module-BR23 and Prostate Module-PR25), pain intensity-NRS), Beck Depression Inventory και Self-Rating Anxiety Scale, SAS), συνεντεύξεων (βλέπε παράρτημα) και αναλύσεων δειγμάτων σάλιου που θα ληφθούν από τους συμμετέχοντες (SALIVARY CORTISOL, SALIVARY α-AMYLASE).

17. **Στατιστική ανάλυση:** Η στατιστική ανάλυση θα γίνει από στατιστικολόγο και πέραν από την περιγραφική ανάλυση θα περιλαμβάνει και επαγωγική ανάλυση. Η περιγραφική στατιστική ανάλυση θα περιλαμβάνει μεθόδους για οργάνωση και περίληψη μιας σειράς δεδομένων με εύκολο και σύντομο τρόπο μέσω πινάκων (π.χ πίνακας συχνοτήτων, σχετική συχνότητα), γραφημάτων και προσδιορισμού μιας ή περισσοτέρων αντιπροσωπευτικών τιμών (π.χ. δειγματική μέση τιμή, δειγματική τυπική απόκλιση). Η επαγωγική στατιστική αποτελεί μία σειρά μεθόδων η οποία χρησιμοποιείται για την εξαγωγή συμπερασμάτων σχετικά με τα χαρακτηριστικά του πληθυσμού βασισμένα σε δεδομένα από το δείγμα. Για τους σκοπούς του προγράμματος θα μελετηθούν μεταξύ άλλων η απλή ανάλυση διασποράς (ANOVA), και t στατιστικό τεστ με ζευγαρωμένα υποκείμενα (paired t tests). Για την ανάλυση των ποιοτικών δεδομένων που θα προκύψουν από τις

συνεντεύξεις θα χρησιμοποιηθεί η τεχνική της «ανάλυσης περιεχομένου». Η *‘ανάλυση περιεχομένου’* είναι μία διαδικασία για την κατηγοριοποίηση των λεκτικών δεδομένων ή των δεδομένων που προκύπτουν από τη συμπεριφορά των ατόμων που συμμετέχουν στη συζήτηση, με σκοπό την ταξινόμηση, τη συνάθροιση και τη διάταξη τους. Το περιεχόμενο μπορεί να αναλυθεί σε δύο επίπεδα. Το βασικό επίπεδο της ανάλυσης είναι ένας περιγραφικός απολογισμός των δεδομένων. Το υψηλότερο επίπεδο της ανάλυσης είναι ερμηνευτικό: έχει να κάνει με το τι συνάγεται και τι υπονοείται. Μερικές φορές ονομάζεται το αφανές επίπεδο της ανάλυσης

18. **Συνειδητή συναίνεση για συμμέτοχη στο Πρόγραμμα:** Οι συμμετέχοντες θα κληθούν να υπογράψουν φόρμα συγκατάθεσης αφού πρώτα ενημερωθούν για τον σκοπό, τις διαδικασίες και τις μεθόδους, τα δικαιώματα που απορρέουν από την συμμετοχή τους αλλά και τις αναμενόμενες από αυτούς ενέργειες. Θα τονιστεί το δικαίωμα των συμμετεχόντων για αποχώρηση από το πρόγραμμα σε οποιαδήποτε στιγμή το θεωρήσουν αυτοί αναγκαίο, χωρίς να υπάρχουν οποιεσδήποτε επιπτώσεις στην φροντίδα που τους παρέχεται άμεσα ή μελλοντικά.

19. **Την ευθύνη για το πρόγραμμα θα την φέρει ο Επιστημονικός Υπεύθυνος**

20. ΔΕΝ ΙΣΧΥΕΙ

21. ΔΕΝ ΙΣΧΥΕΙ (χρήση γενετικών δεδομένων)

22. **Τα προσωπικά δεδομένα που θα συνοδεύουν το δείγμα του πληθυσμού του προγράμματος – ΔΕΝ ΙΣΧΥΕΙ –** Το δείγμα θα συνοδεύεται μόνο από γενικά δημογραφικά στοιχεία.

23. **Τα δημογραφικά δεδομένα που θα συνοδεύουν το δείγμα του πληθυσμού του προγράμματος θα περιλαμβάνουν την επαρχία διαμονής, το φύλο, ηλικία, χρόνος διάγνωσης, τύπος καρκίνου.**

24. **Διάχυση προσωπικών δεδομένων –** ΔΕΝ ΙΣΧΥΕΙ

25. **Διάχυση γενετικών δεδομένων –** ΔΕΝ ΙΣΧΥΕΙ

26. **Διάχυση δειγμάτων γενετικού υλικού ή άλλων βιολογικών δειγμάτων - ΔΕΝ ΙΣΧΥΕΙ –** Μετά τις απαραίτητες εργαστηριακές εξετάσεις (αμυλάση α και κορτιζόλη σάλιου) τα δείγματα θα καταστραφούν.

27. **Πρόσβαση σε πληροφορίες από τους συμμετέχοντες στο πρόγραμμα:** Όλα τα αποτελέσματα και πληροφορίες του προγράμματος θα είναι διαθέσιμα σε όλους τους συμμετέχοντες στο πρόγραμμα και στους συγγενείς α’ βαθμού (σύζυγος, παιδιά).

28. **Χρόνος αποθήκευσης και καταστροφής δειγμάτων και δεδομένων:** Η αποθήκευση των δειγμάτων θα γίνει για το ελάχιστο χρονικό διάστημα που

απαιτείται για την εργαστηριακή εξέταση των δειγμάτων. Κάθε δείγμα θα εξετάζεται άμεσα και θα αποφεύγεται η αποθήκευση του εκεί και όπου υπάρχει η δυνατότητα. Με το πέρας του προγράμματος όλα τα δείγματα θα καταστραφούν με τον ενδεδειγμένο τρόπο (πρωτόκολλο) που επιβάλλεται για την καταστροφή βιολογικών δειγμάτων.

29. **Διαδικασία υποβολής παραπόνων ή καταγγελιών:** Η υποβολή τυχόν παραπόνων ή καταγγελιών θα μπορεί να γίνει άμεσα στον υπεύθυνο έρευνας του φορέα στον οποίο διεξάγεται το ερευνητικό πρόγραμμα. Στο συγκεκριμένο πρόγραμμα, η υποβολή των παραπόνων θα γίνεται άμεσα στον Δρ Χαράλαμπο Χρυσοστόμου, Προϊστάμενο Υπηρεσίας ‘Ερευνας Διεθνών και Δημοσίων Σχέσεων (ΥΕΔΔΣ) – ΤΕΠΑΚ.

    Στοιχεία Επικοινωνίας

    Τεχνολογικό Πανεπιστήμιο Κύπρου

    Αθηνών 80 ΤΤ 3036 Λεμεσός

    Τηλ 00357 25002562 Fax 0035725002763

    Ηλεκτρονικό Ταχυδρομείο c.chrisostomou@cut.ac.cy

Επιλεγμένες Παραπομπές

Eisenberg DM, Davis RB, Ettner SL, et al. Trends in alternative medicine use in the United States, 1990–97: results of a follow-up national survey. JAMA. 1998;280:1569–75.

Wootton JC, Sparber A. Surveys of complementary and alternative medicine: part I. General trends and demographic groups. J Altern Complement Med. 2001;7:195–208.

Kiecolt-Glaser JK, Mcguire L, Robles TF, Glaser R. Psychoneuroimmunology and psychosomatic medicine: back to the future. Psychosom Med. 2002;64:15–28.

Stefano GB, Fricchione GL, Slingsby BT, Benson H. The placebo effect and relaxation response: neural processes and their coupling to constitutive nitric oxide. Brain Res Rev. 2001;35:1–19.

Sobel DS. Mind matters, money matters: the cost-effectiveness of mind-body medicine. JAMA. 2000;284:1705.

Sobel DS. The cost-effectiveness of mind-body medicine interventions. Prog Brain Res. 2000;122:393–412.

Friedman R, Sobel D, Myers P, Caudill M, Benson H. Behavioral medicine, clinical health psychology, and cost offset. Health Psychol. 1995;14:509–18.

MacLennan AH, Wilson DH, Taylor AW: The escalating cost and prevalence of alternative medicine. Preventive Medicine 2002, 35:166-173.

Eisenberg DM, Davis RB, Ettner SL, Appel S, Wilkey S, Van Rompay M, Kessler RC: Trends in alternative medicine use in the United States, 1990-1997. JAMA 1998, 280:1569-1515.

White AR, Ernst E: Economic analysis of complementary medicine: a systematic review. Complementary Therapies in Medicine 2000, 8:111-118.

Pelletier KR, Astin JA: Integration and reimbursement of complementary and alternative medicine by managed care and insurance providers: 2000 update and cohort analysis. Alternative Therapies 2002, 8:38-48.

Neumann PJ: Evidence-based and value-based formulary guidelines. Health Affairs 2004, 23:124-134.

Sullivan SD, Lyles A, Luce B, Grigar J: AMCP guidance for submission of clinical and economic evaluation data to support formulary listing in US health plans and pharmacy

benefits management organizations. Journal of Managed Care Pharmacy 2001, 7:272-282.

Claxton K, Neumann PJ, Araki S, Weinstein MC: Bayesian value-of information analysis: an application to a policy model of Alzheimer's disease. International Journal of Technology Assessment in Health Care 2001, 17:38-55.

Claxton K, Posnett J: An economic approach to clinical trial design and research priority-setting. Medical Economics 1996, 5:513-524.

Παραρτήματα

Σχεδιασμός της έρευνας

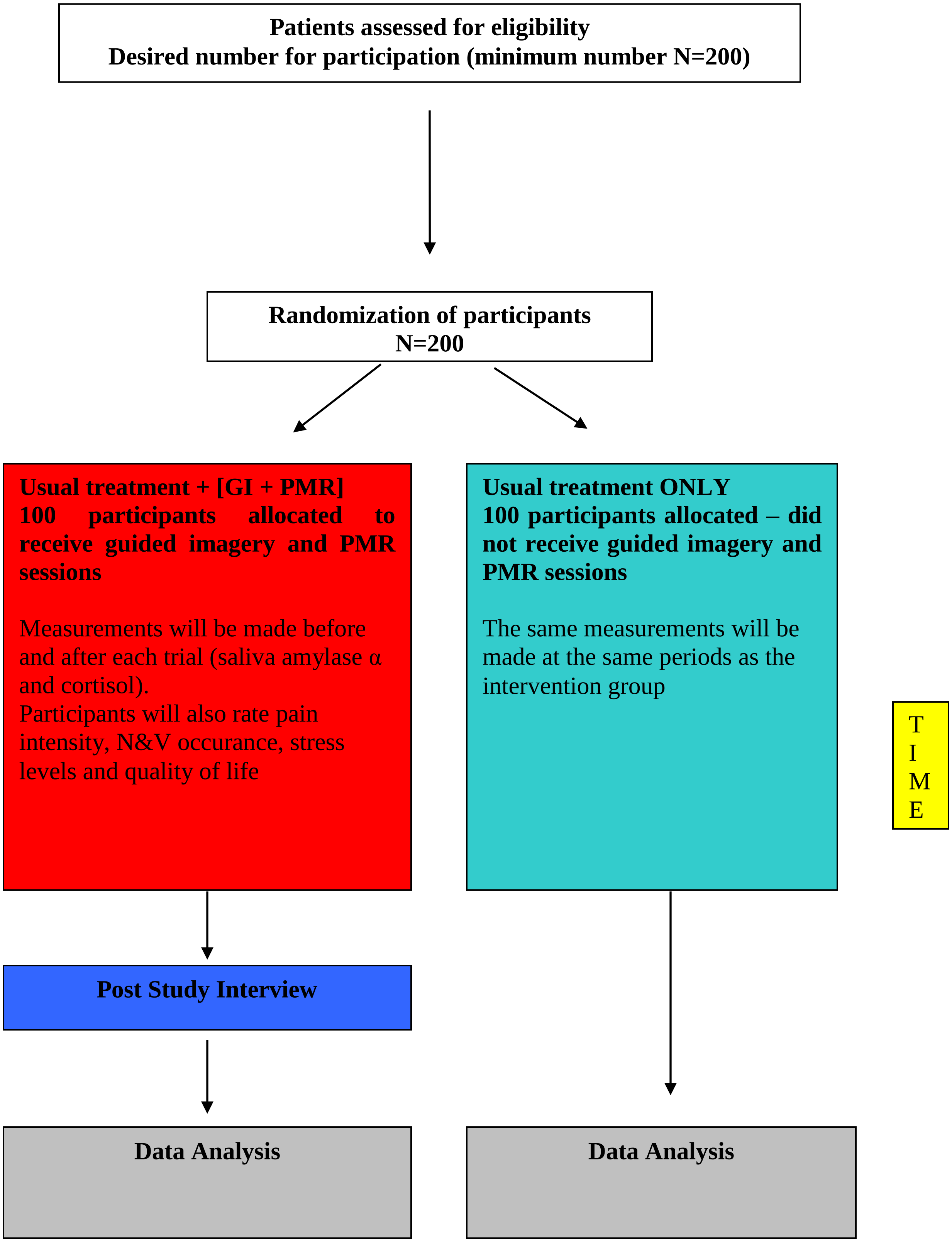

Table 1 Ερωτήσεις Συνέντευξης

**Τι πιστεύεται για τις τεχνικές του κατευθυνόμενου οραματισμού και της προοδευτικής μυϊκής χαλάρωσης ?**<br><br>**1. Το απολαύσατε?**<br><br>**2. Πιστεύετε ότι έχουν δουλέψει για εσάς?**<br>**α. Αν όχι: Έχετε κάποια ιδέα για τους λόγους που δεν δούλεψαν για σας?**<br>**β. Αν ναι: Έχετε κάποια ιδέα για τους λόγους που δούλεψαν για σας?**<br>**Πόσο κράτησε η επίδραση τους για σας?**<br><br>**3. Ήταν η διάρκεια τους ικανοποιητική? Θα προτιμούσατε μεγαλύτερη, μικρότερη, ή την ίδια?**<br><br>**4. Νιώθετε ότι δοκιμάζοντας τις τεχνικές 4 φορές κατω απο καθοδήγηση ήταν αρκετές για να διαπιστώσετε αν ήταν βοηθητικές για σας ή όχι?**
